# Optimizing Detection Reliability in Safety-Critical Computer Vision: Transfer Learning and Hyperparameter Tuning with Multi-Task Learning

**DOI:** 10.3390/s25206306

**Published:** 2025-10-12

**Authors:** Waun Broderick, Sabine McConnell

**Affiliations:** Department of Computer Science, Trent University, Peterborough, ON K9L 0G2, Canada; sabinemcconnell@trentu.ca

**Keywords:** landmine detection, computer vision, multi-task learning, transfer learning, hyperparameter tuning, thermal imaging, humanitarian demining, safety-critical systems, object detection, deep learning

## Abstract

This paper presents a methodological framework for selectively optimizing computer vision models for safety-critical applications. Through systematic processes of hyperparameter tuning alongside multitask learning, we attempt to create a highly interpretable system to better assess the dangers of models intended for safety operations and intentionally select their trade-offs. Using thermographic images of a specific imitation explosive, we create a case study for the viability of humanitarian demining operations. We hope to demonstrate how this approach provides a developmental framework for creating humanitarian AI systems that optimize safety verification in real-world scenarios. By employing a comprehensive grid search across 64 model configurations to evaluate how loss function weights impact detection reliability, with particular focus on minimizing false negative rates due to their operational impact. The optimized configuration achieves a 37.5% reduction in false negatives while improving precision by 2.8%, resulting in 90% detection accuracy with 92% precision. However, to expand the generalizability of this model, we hope to call institutions to openly share their data to increase the breadth of imitation landmines and terrain data to train models from.

## 1. Introduction

The global impact of landmines and unexploded ordnance (UXO) remains a persistent humanitarian challenge despite international efforts to ban their use. According to the Landmine Monitor 2022 report, at least 5544 casualties from mines and explosive remnants of war were recorded in 2021 alone, with civilians accounting for 80% of all casualties [1]. More recent data from the Landmine Monitor 2023 has shown the continued threat these devices pose to civilian populations worldwide [2]. According to recent data from the United Nations Mine Action Service (UNMAS), over 60 countries and territories are still contaminated with landmines, affecting the lives and livelihoods of millions of people worldwide.

While significant progress has been made in detecting the presence of UXOs, creating models that can be employed to effectively localize exact areas of landmines, while communicating their alignment with safety standards, is still underway. A distinction that is particularly important in areas with high UXO density, complex terrain, and in inhabited areas.

With the rapid expansion of drone technology, research has increased the capabilities of drone-based sensing methods. The commercialization of many of its new applications within fields such as entertainment, agriculture, and defense has created a scalable economy to drive down its associated costs, making it a highly viable solution vector.

This is particularly important due to the diversity of landmine employment methods and physical types. From the materials to above-ground or in-ground implementations, a solution would need to take into account a breadth of variables. Thermal imaging, in particular, offers unique advantages through its ability to detect subtle temperature differences caused by buried objects with thermal properties distinct from those of the surrounding soil, a characteristic similar to UXOs, due to their reliance on thermoactive chemical compounds within their payloads.

The intersection between low-cost drones’ availability and advancements in thermo-graphic imaging with ML models creates a great opportunity for integration into humanitarian demining operations. An ability to lower the current standard of false-positive landmine detections, not just a statistical milestone, but the ability to improve the safety of civilian lives; children returning to schools, farmers being able to work their land, and the ability for communities to begin to truly rebuild.

## 2. Literature Review

### 2.1. Drone-Based Systems for Landmine Detection

Early explorations into utilizing drones for demining operations have been explored by researchers like Kowalski et al. [3] who conducted a detailed investigation of drone systems for detecting explosive hazards, highlighting the strengths of aerial platforms in covering large areas efficiently while maintaining a safe distance from potential threats. The research showed the viability of sensor integration with real-time processing for developing effective drone-based detection systems, though it also highlighted a gap in localization precision that our study hoped to address due to its importance when it comes to the specific removal of UXOs.

Some areas that still contain UXOs are also those that are less inhabited due to a lack of infrastructure or difficult terrain, though, are still used by many people. Drones also offer a particular advantage in these post-conflict zones as explored by Luna et al. [4] who observed that UAVs provide access to dangerous or inaccessible areas for human operators, which could potentially speed up demining operations while substantially reducing risks. Their system showed the feasibility of integrating multiple sensing technologies on a single drone platform, creating a more comprehensive detection capability.

### 2.2. Methodologies for Subsurface Landmine Detection

As conflicts change and evolve over time, landmines also innovate and become more dangerous and difficult to detect. Challenges in keeping up with the changing technology of buried landmines have led to the development of various methodologies with differing sensing methods. Makki et al. [5] detail a comparative analysis of multisensor approaches for landmine detection, examining ground-penetrating radar (GPR), magnetometry, infrared thermography, and other techniques. Their analysis revealed that each modality has specific strengths and limitations depending on environmental conditions, mine composition, and burial depth, though no ‘one size fits all’ solution.

### 2.3. Thermographic Imaging for Landmine Detection

While many sensing methods have focused on metallic mines, as time has progressed, people have found more ways to minimize the amount of metals within a landmine’s construction to avoid detection. Thermographic imaging offers distinct advantages, particularly for detecting both metallic and non-metallic explosive devices by registering the differences in the thermal capacity between the explosive chemical compounds and their surroundings. Santulli [6] found in his review of infrared thermography for buried object detection that thermal imaging can identify temperature differences between the soil and buried objects resulting from their varying thermal properties, a capability that is particularly valuable for detecting modern landmines that may intentionally contain minimal metallic components. To maximize the difference between these capacities, taking into account environmental changes and states would be very important as determined by a study conducted by Doe and Williams [7]. They determined that infrared thermography performs especially well during daily temperature transitions when buried objects and the surrounding soil exhibit maximum thermal contrast. Their research found that thermography can detect both surface and shallow-buried landmines with higher reliability than GPR in dry, sandy soils where radar signals may experience significant degradation in their efficacy, which is a valuable point to consider for data diversification and real-world employment.

### 2.4. Deep Learning Approaches for Landmine Detection

In recent years, significant advancements have been made in pairing new sensing methods to deep learning techniques for the challenge of landmine detection. Foundational sensors and methodologies have been explored by researchers like Safatly et al. [8] who successfully achieved classification accuracies of over 85% for differentiating between landmines and metallic debris when using classification models with metal landmine detectors. Their approach, while limited to metallic objects, starts to explore the integration of models that can be trained to optimize for selective tuning like the lowering of false negatives.

Further advancing the application of deep learning in this domain, Vivoli et al. [9] implemented a real-time detection system for surface landmines using optical imaging. Their approach employed YOLOv5, demonstrating the efficacy of the YOLO (You Only Look Once) architecture family for object detection in the context of explosives detection. The authors found that their system achieved real-time processing capabilities with detection accuracies exceeding 90% under favorable lighting conditions, establishing an important benchmark for detection speed and accuracy in above-ground field deployments.

### 2.5. Multi-Task Learning for Enhanced Object Detection

Multi-task learning (MTL) represents a paradigm where a single neural network simultaneously learns multiple related tasks, leveraging shared representations to improve performance across all objectives. In the context of object detection, MTL approaches can simultaneously optimize for classification, localization, and auxiliary tasks such as depth estimation or semantic segmentation. Zhang and Yang [10] provided a comprehensive survey of multi-task learning methodologies, demonstrating that shared feature representations learned across related tasks often lead to improved generalization performance compared to single-task approaches, particularly when training data is limited.

The application of multi-task learning to object detection has shown particular promise in scenarios where multiple types of information must be extracted from the same input. Ruder [11] observed that MTL can act as a form of regularization, reducing overfitting by encouraging the model to learn more general representations that are useful across multiple tasks. In the future expansion of this model, where different signals within terrain and landmine characteristics are especially of interest to be able to separate and discern, this becomes a very important point for optimization.

The quickly advancing field of multi-task learning for computer vision has shown the effectiveness of hard parameter sharing architectures, where early layers of the network are shared across tasks while task-specific layers handle specialized processing. Kendall et al. [12] showed that the careful weighting of multiple loss functions in MTL frameworks can significantly improve performance on individual tasks compared to single-task baselines, establishing important principles for designing effective multi-task detection systems in safety-critical applications such as humanitarian demining.

### 2.6. Evolution of YOLO Architectures for Object Detection

The YOLO family of object detection architectures has evolved significantly since its introduction, with each iteration bringing improvements in accuracy, speed, and feature extraction capabilities. Redmon and Farhadi [13] introduced YOLOv3, which incorporated residual networks and feature pyramid networks to improve detection accuracy across objects of varying scales. The chosen architecture revealed particular strength in detecting small objects, a critical capability for landmine detection applications where targets may occupy a small portion of the image frame.

Wang et al. [14] further advanced the YOLO architecture with YOLOv7, which introduced trainable bag-of-freebies techniques that substantially improved model performance without increasing inference costs. Their architecture achieved state-of-the-art results on the MS COCO dataset [15], establishing new benchmarks for real-time object detection performance.

The latest evolution in this architectural family, YOLOv8, builds upon these advances with significant improvements in backbone design, loss function optimization, and hyperparameter sensitivity. According to Jocher et al.’s [16] seminal paper, YOLOv8 features a more efficient backbone, improved path aggregation neck, and anchor-free detection heads that enhance both accuracy and inference speed compared to previous versions. These architectural improvements make YOLOv8 a particularly promising candidate for landmine localization applications where both precision and computational efficiency are critical requirements.

### 2.7. Transfer Learning for Specialized Detection Tasks

Given the scarcity of large-scale labeled datasets in the landmine detection domain, transfer learning approaches offer substantial advantages by leveraging knowledge gained from models pre-trained on large general-purpose datasets. Mahmoud, K.A.A [17] found that models previously trained on natural image datasets could be effectively adapted to thermal imagery classification tasks using relatively small amounts of domain-specific training data.

Rajagopalan et al. [18] further explored the application of transfer learning techniques to infrared image object detection, concluding that kernel-based transfer learning methods can effectively connect the domains of visible and infrared imagery, allowing more efficient model training for specialized detection tasks. Their research highlighted the importance of careful feature extraction and adaptation when transferring learned representations between different imaging methods.

The application of transfer learning to explosive hazard detection has been further explored by Zhang et al. [19] in their analysis of different multi-modal deep learning approaches. The researchers found that transfer learning provides more robust feature extraction by incorporating knowledge from diverse datasets, potentially improving adaptation to the varied environmental conditions encountered in real-world demining operations. This observation is particularly relevant for landmine detection systems that must operate reliably across different soil types, moisture levels, and diurnal temperature variations. Due to the lack of diverse landmine data available at the time of this study, this should serve as a promising future possibility if datasets can be expanded.

### 2.8. Research Gap and Proposed Approach

While previous studies have demonstrated the potential of deep learning for landmine detection using various imaging methods, several key research gaps remain. Many existing approaches have focused on detection (determining presence) rather than precise localization (determining exact coordinates), which is critical for efficient clearance operations. The applications of transfer learning and hyperparameter optimization specifically for thermographic landmine imagery remain relatively unexplored, particularly with the latest YOLO architectures. Joining these two opportunities to work towards building an interpretable model that can articulate safety tradeoffs is also of the highest importance. Given that our dataset only contains a single imitation legbreaker mine type and a lack of diverse environmental implementations, the ability to generalize would be low when applied to diverse operational environments.

Though we hope to build this future framework by leveraging pre-trained weights from large general-purpose datasets and systematically optimizing hyperparameters for the specific characteristics of thermal landmine signatures, this approach aims to achieve precise localization while maintaining model efficiency suitable for deployment on drone platforms with limited processing resources.

## 3. Data

### 3.1. Pretraining Dataset

Ultralytic’s Yolov8 model builds upon previous YOLO architectures utilizing a combination of loss functions for fast and efficient object detection. It was primarily pre-trained on the Microsoft Common Objects in Context (COCO) dataset [15], an undertaking facilitated by a team comprising researchers from Microsoft Research, Cornell University, and Caltech. COCO is a large database of images containing several objects that are precisely pixel-level annotated to be used to train computer vision models.

The COCO dataset contains over 330,000 images with more than 2.5 million labeled instances across 80 common object categories. These categories contain a diverse breadth of objects commonly encountered in life, making the dataset particularly capable in transfer learning for general object detection tasks [15]. At the time of this paper, COCO is still one of the leading open source datasets for computer vision models.

Since YOLOv8 allows us to leverage its transfer learning, it significantly reduces the need for an extensive repository of visual data. By utilizing YOLOv8’s generalized visual encodings foundation, we hope to adapt the model to our specialized domain despite our relatively small custom dataset. If this is successful, we can greatly accelerate convergence, and achieve high degrees of accuracy in our detection.

### 3.2. Landmine Dataset

The dataset used for this study contains 2700 unique thermographic images of terrain with buried antipersonnel “legbreaker” landmines. These images were acquired at different heights using a Zenmuse XT infrared camera (7–13 µm) embedded in the DJI Matrice 100 drone [20]. The landmines were planted in the ground at varying depths (0 cm, 1 cm, 5 cm, and 10 cm) and spread over an area of 10 m × 10 m. The dataset includes images in JPG, TIFF, and RJPG formats.

The experimental setup involved a systematic protocol to capture thermal images under specific conditions. Following recommendations from previous studies, image acquisition was performed on sunny, cloudless days between 17:00 and 18:00 h, when thermal differences between the ground and antipersonnel mines are most pronounced. Environmental conditions were carefully monitored, with optimal wind speeds of 3–5 m/s and ambient temperatures ranging from 26 to 30 °C to favor thermal diffusion between the mines and ground surface [20].

The terrain used in the experiment featured non-saline soil with a pH of 5.73 (moderately acidic), 35.6% sand, 35.4% clay, and 29% silt composition. Images were captured at altitudes ranging from 1 to 10 m (at 1 m intervals) to evaluate the effect of spatial resolution on detection capabilities. At each height, multiple images were captured to account for variability due to drone positioning and thermal camera noise [20].

### 3.3. Characteristics of the Imitation Landmines

**Figure 1:**
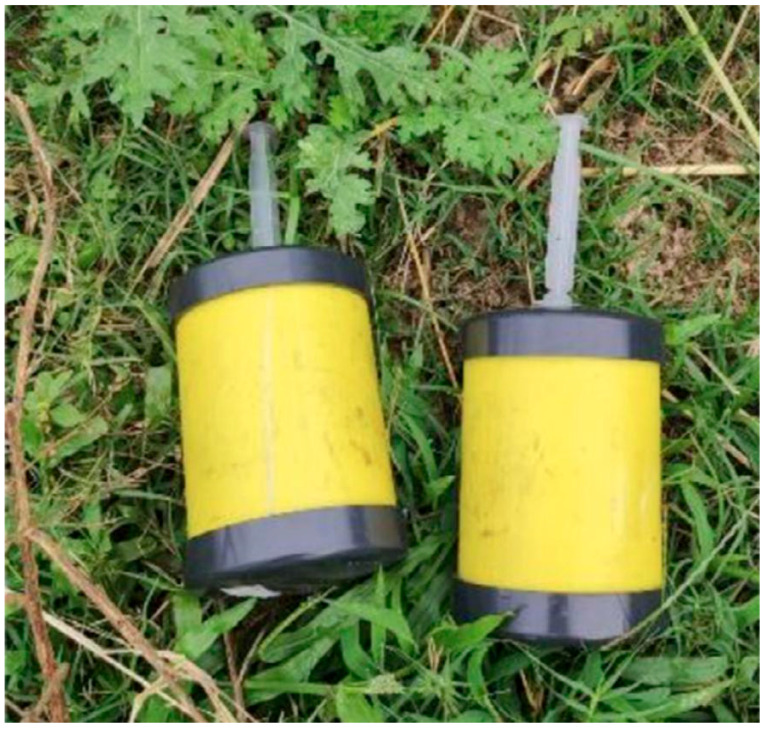
Imitation antipersonnel landmines constructed for thermal imaging experiments. Each device consists of a PVC cylinder (8.7 cm diameter, 10.2 cm height) filled with anthracite coal to simulate TNT thermal properties, with a 5 mL syringe simulating the detonator mechanism. The yellow casing provides visual contrast while maintaining thermal signature authenticity for safe experimental validation [20].

The landmines used in this dataset (Figure 1) were specifically designed to simulate the thermal properties of actual antipersonnel “legbreaker” mines. Each imitation mine consists of a PVC cylinder with dimensions matching those established by the Colombian national army (8.7 cm diameter, 10.2 cm height, and 1.4 cm plunger diameter). The mines were filled with anthracite coal, which has thermal characteristics similar to those of TNT, with minimal differences in specific heat (0.11 J/g·K) and thermal conductivity (0.03 W/m·K) [20].

This choice of materials is particularly relevant as modern landmines increasingly use polymer-based components rather than traditional metal casings. The thermal properties of these polymers create distinctive thermal signatures that can be captured through infrared imaging, especially during periods of thermal transition in the environment. The simulation of these modern threats by employing imitation landmines with similar thermal compositions makes the research directly applicable to ongoing humanitarian demining efforts.

**Figure 2:**
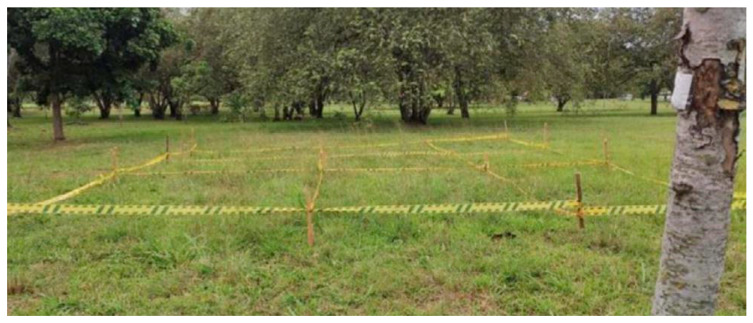
Controlled experimental test site for thermal landmine signature acquisition. The 10 m × 10 m area contains nine marked zones with imitation landmines buried at depths of 0, 1, 5, and 10 cm, plus one mine-free control zone. Yellow boundary markers enable precise spatial registration for drone-based thermal imaging validation. Site selection criteria included non-saline soil composition (pH 5.73) and minimal vegetation interference to maximize thermal contrast during optimal acquisition windows (17:00–18:00 h).

The use of anthracite coal instead of actual explosives enabled safe experimentation while maintaining the thermal validity of the detection approach. Each mine also incorporated a 5 mL syringe to simulate the detonator mechanism, with the plunger partially visible at the surface for mines at 0 cm depth but hidden by vegetation for buried mines as seen in (Figure 2), demonstrating the advantage of thermal imaging over visible spectrum detection.

### 3.4. Multi-Task Learning Architecture

Multi-task learning in object detection involves simultaneously optimizing multiple related objectives within a single neural network architecture. In the context of YOLOv8 for landmine detection, this approach leverages shared feature representations to jointly learn classification, localization, and coordinate regression tasks.

**Figure 3:**
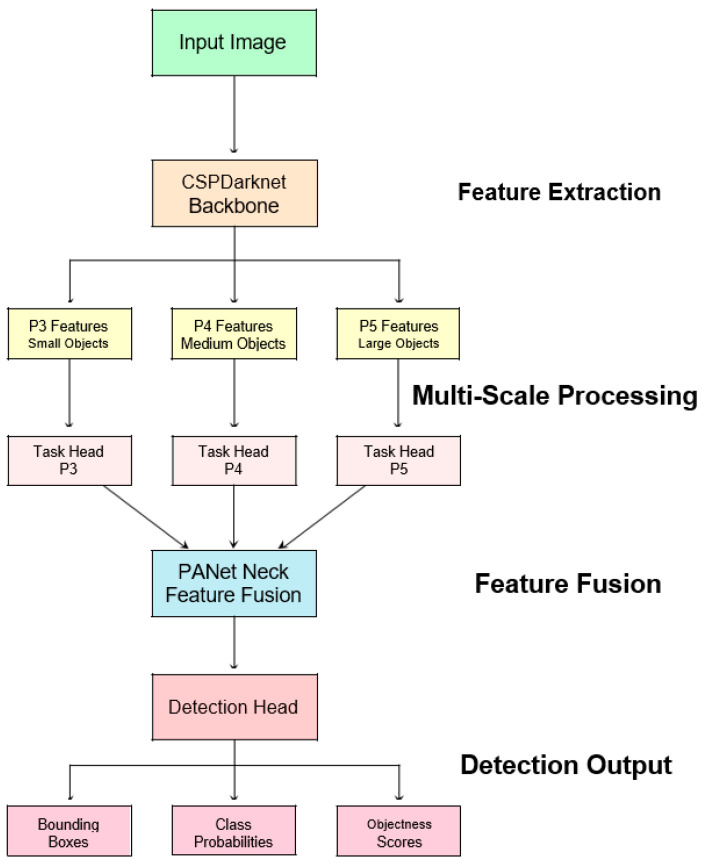
YOLOv8 multi-task learning architecture adapted for landmine detection processes thermal images through three sequential stages: (1) CSPDarknet backbone performs feature extraction to construct hierarchical representations from raw thermal imagery; (2) multi-scale feature pyramid heads (P3-P5) detect landmine signatures at different burial depths and altitudes; (3) task-specific detection heads simultaneously optimize three objectives using weighted loss functions (*λ*_cls_ for classification confidence, *λ*_box_ for spatial localization, *λ*_dfl_ for coordinate precision). This parameter sharing approach enables the model to computationally weigh commonalities between detection tasks while maintaining specialized processing for our specific landmine searching use case.

The multi-task learning framework in YOLOv8 (Figure 3) employs hard parameter sharing, where early layers learn shared representations across all tasks, while task-specific heads handle specialized processing for classification, localization, and objectness prediction. This approach enables the model to leverage commonalities between related tasks while maintaining task-specific expertise in the final layers.

### 3.5. Multi-Task Loss Function Optimization

The effectiveness of multi-task learning depends critically on the proper weighting and combination of task-specific loss functions. YOLOv8 implements a composite loss function that balances three complementary objectives: spatial localization accuracy, classification confidence, and coordinate precision.

### 3.6. Localization Task Loss (L_loc_)

The localization task focuses on accurately predicting bounding box coordinates using Complete IoU (CIoU) loss:

$$L_{\mathrm{loc}\;}=1-CIoU=1-(IoU-\frac{\rho^{2}(\;b,{\;b}^{gt})}{c^{2}}-\frac{v^{2}}{(1-IoU)+v^{2}})$$

where IoU measures spatial overlap, *ρ*(b, b^gt^) captures center point distance, and v enforces aspect ratio consistency:

$$v=\frac{4}{\pi^{2}}{\left(arctan\frac{w^{gt}}{h^{gt}}-arctan\frac{w}{h}\right)}^{2}$$

This multi-component loss simultaneously optimizes multiple geometric relationships, demonstrating the multi-task nature even within individual loss components.

### 3.7. Classification Task Loss (L_cls_)

The classification task employs Binary Cross-Entropy (BCE) loss to optimize class prediction accuracy:

$$L_{cls}=-\frac{1}{N}\sum_{i=1}^{N}\sum_{c=1}^{C}\left[y_{i,c}log\left(p_{i,c}\right)+(1-y_{i,c})log(1-\;p_{i,c})\right]$$

where N represents predicted objects, C denotes classes, y_i,c_ indicates ground truth labels, and p_i,c_ represents predicted probabilities. This task benefits from shared feature representations learned through the localization task.

### 3.8. Coordinate Regression Task (DFL)

The Distribution Focal Loss (DFL) represents a specialized regression task that models coordinate uncertainty through distributional predictions:

$$L_{\mathrm{dfl}}=-\sum_{i=1}^{n}\;\sum_{j=1}^{d}y_{i,j}log(p_{i,j})$$

where n represents coordinates, d denotes discretization bins, and the final coordinate value emerges as

$$\hat{v}=\sum_{j=1}^{d}j\cdot p_{j}$$

This probabilistic approach to coordinate regression exemplifies how multi-task learning can enhance individual task performance through sophisticated output representations.

### 3.9. Multi-Task Loss Integration

The multi-task learning framework combines all task-specific losses through weighted summation:

*L_total_* = *λ_loc_* · *L_loc_* + *λ_cls_* · *L_cls_* + *λ_dfl_* · *L_dfl_*

The weighting parameters λloc, λcls, and λdfl control the relative importance of each task, enabling the dynamic balancing of multi-task objectives based on application requirements.

## 4. Grid Search Methodology

### 4.1. Model Architecture Modifications

To optimize our YOLOv8 model for the specific requirements of demining operations, we implemented architectural modifications to the base model. The final layer of the network was adjusted to focus specifically on mine detection. The modification reconfigured the model to output a single class prediction, optimizing the network specifically for mine detection rather than multi-class object detection.

### 4.2. Hyperparameter Grid Search

When dealing with the realities of landmine hunting, the desire to use an algorithm with a high level of interoperability was one of the main concerns. Depending on the operation, the level of resources and processes may warrant different tolerances to certain metrics. This methodology allows us to visually understand, weigh, and question the real tradeoffs individually and with intention.

#### 4.2.1. Search Space Definition

The grid search implementation explored combinations of the three primary loss function weights in the YOLOv8 architecture:

This resulted in a total of 64 distinct model configurations (4 box weight values × 4 class weight values × 4 DFL weight values) (Table 1).

#### 4.2.2. Training Protocol

Each of the 64 model variants was trained using a standardized protocol:

- K-Fold Cross-Validation: Each model was trained using 5-fold cross-validation with shuffling enabled and a fixed random state to ensure reproducibility.
- Consistent Data Splitting: Training and validation data directories were specified through YAML configurations for each fold.
- Hardware Configuration: All models were trained on identical hardware configurations to eliminate variability.
- Training Duration: Each model was trained for 100 epochs with an early stopping patience of 20 epochs.
- Batch Size: A consistent batch size of 16 was used across all training runs.
- Optimizer: All models used the AdamW optimizer with an initial learning rate of 0.001 and cosine learning rate scheduling.

### 4.3. Evaluation Framework

#### 4.3.1. Performance Metrics

Each model variant was evaluated using a comprehensive set of metrics:

- Mean Average Precision (mAP): Standard detection performance at an IoU threshold of 0.5.
- Precision: The ratio of true positive detections to all positive detections.
- Recall: The ratio of true positive detections to all ground truth objects.
- False Negative Rate (FN Rate): The proportion of mines that went undetected, calculated as 1 – Recall.
- Balanced Score: A composite metric calculated as mAP × Recall to identify models with good overall performance while maintaining high recall.

#### 4.3.2. Statistical Analysis

For each model configuration, we aggregated performance metrics across all five folds to calculate:

- Mean performance values
- Standard deviations to assess stability
- 95% confidence intervals for robust comparison.

#### 4.3.3. Visual Analysis

We implemented comprehensive visualization techniques to analyze the grid search results:

- Parameter Response Curves: Visualized the relationship between individual hyper-parameters and performance metrics.
- Heatmaps: Created multi-dimensional visualizations to identify interactions between pairs of hyperparameters while holding the third constant.
- 3D Scatter Plots: Plotted the entire parameter space with color-coding by performance metrics.
- Trade-off Analysis: Visualized the relationship between false negative rate and precision to identify optimal trade-off points.

## 5. Results

The chosen grid search of 64 hyperparameter configurations yielded significant in-sights into the optimal settings for mine detection with YOLOv8. Through systematic testing and analysis, we identified clear parameter effects on key performance metrics and their trade-offs.

### 5.1. Hyperparameter Effects on Performance Metrics

#### 5.1.1. Effects on False Negative Rate

**Figure 4:**
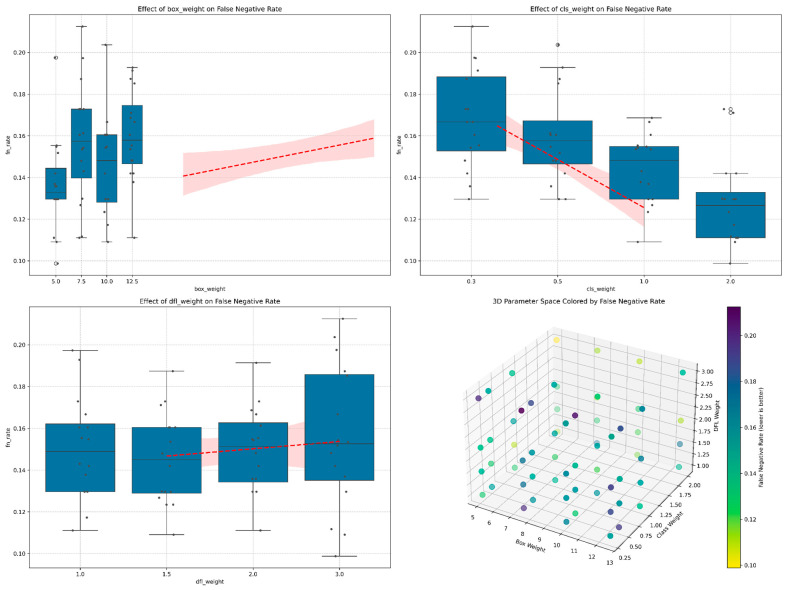
Hyperparameter sensitivity analysis for false negative rate optimization in landmine detection. (**Top panels**) Individual parameter effects showing class weight exhibits strongest inverse correlation (23.5% improvement from *λ*_cls_ = 0.3 to 2.0), while box weight demonstrates optimal performance at *λ*_box_ = 5.0. (**Bottom right**) 3D parameter space visualization reveals consistent false negative rate reduction (blue regions) with high class weights and moderate box weights, critical for safety-critical applications.

The false negative rate, critical in mine detection operations, showed strong dependence on hyperparameter settings:

- Class Loss Weight (λ_cls_): Exhibited the strongest influence on false negative rate, with a clear inverse relationship. As shown in Figure 4, increasing the class loss weight from 0.3 to 2.0 resulted in a substantial and consistent reduction in false negative rate from a median of approximately 0.17 to 0.13. This represents a relative improvement of approximately 23.5%.
- Box Loss Weight (*λ*_box_): Demonstrated a non-monotonic relationship with a false negative rate, with the lowest FN rates observed at box weight values of 5.0. Increasing box weight beyond this value generally led to higher false negative rates, suggesting that overemphasis on box accuracy may come at the expense of detection completeness.
- DFL Loss Weight (*λ*_dfl_): Showed the weakest correlation with a false negative rate, with a slight upward trend as the weight increased. This indicates that while coordinate precision is important, it has less impact on detection recall than the other parameters.

**Figure 5:**
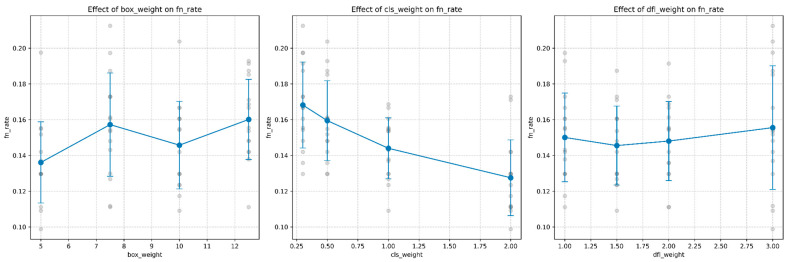
Hyperparameter sensitivity profiles for false negative rate optimization. Class weight (*λ*_c_ls) shows the strongest linear relationship with 95% confidence intervals, indicating reliable performance gains. Box weight exhibits non-monotonic behavior with optimal performance at *λ*_b_ox = 5.0, while DFL weight shows minimal impact on detection completeness. Gray points represent individual fold results across 5-fold cross-validation, demonstrating consistent trends critical for safety-critical parameter selection.

Figure 5 presents the detailed response curves for false negative rate across each parameter dimension, highlighting both individual data points and the statistical trends with confidence intervals.

#### 5.1.2. Effects on Mean Average Precision (mAP)

Model accuracy as measured by mAP@0.5 showed distinct patterns across the parameter space:

- Class Loss Weight: Demonstrated the strongest positive correlation with mAP, with values increasing steadily from 0.52 at *λ*_cls_ = 0.3 to approximately 0.54 at *λ*_cls_ = 2.0. This suggests that increasing emphasis on correct classification directly improves overall detection performance.
- Box Loss Weight: Showed a slight negative trend with mAP, with the highest values observed at the lowest box weight setting of 5.0 (Figure 4). This counter-intuitive result suggests that overemphasizing bounding box accuracy can potentially distract the model from overall detection performance.
- DFL Loss Weight: Had minimal impact on mAP, with a slight negative correlation as weight increased, supporting the finding that coordinate precision plays a secondary role in overall detection performance.

The response curves in Figure 5 further illustrate these relationships, with the positive trend for class weight contrasting with the relatively flat or slightly negative trends for box and DFL weights.

#### 5.1.3. Effects on False Negative Rate and Map

**Figure 6:**
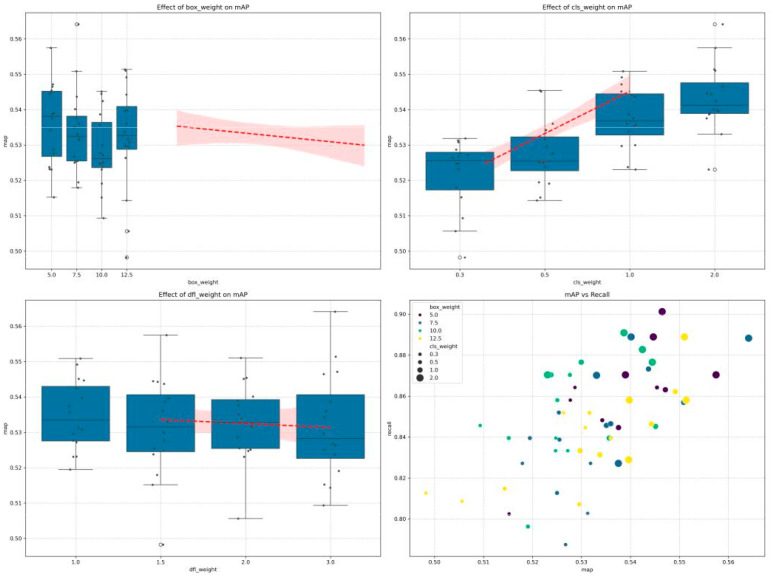
Effects of box weight, class weight, and DFL weight on mean average precision (mAP). The bottom right panel shows the relationship between mAP and recall across different configurations.

**Figure 7:**
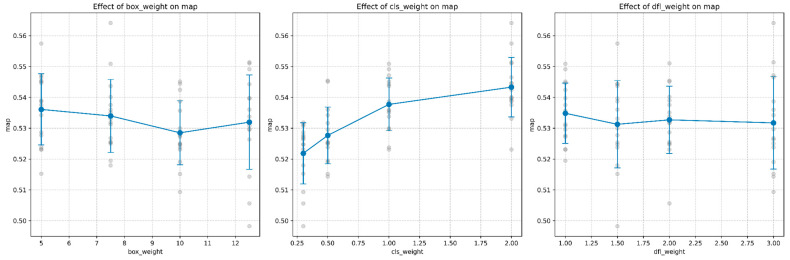
Response curves showing the effect of each hyperparameter on mean average precision (mAP). The clear positive trend for class weight contrasts with the relatively flat or slightly negative trends for box and DFL weights.

Precision and recall metrics revealed additional insights into model behavior:

- False Negative Rate: Class weight demonstrated the strongest effect on false negative rate, showing a clear negative correlation. As shown in Figure 6, increasing class weight from 0.25 to 2.0 reduced the false negative rate from approximately 0.168 to 0.129, representing a relative improvement of 23.2%. Box weight exhibited a non-monotonic relationship, with peak performance (lowest fn_rate of ~0.135) occurring at weight 5, followed by fluctuations at intermediate values. DFL weight showed minimal impact on false negative rate, maintaining relatively consistent values between 0.145 and 0.155 across the tested range.
- Map: The relationship between mAP and the three hyperparameters, shown in Figure 7, revealed that class weight had the most pronounced positive effect. Increasing class weight from 0.25 to 2.0 improved mAP from 0.522 to 0.544, a relative gain of 4.2%. This improvement directly corresponds to the reduction in false negative rate, as higher recall (1 − FN rate) contributes to better overall detection performance. Box weight and DFL weight showed comparatively minor effects on mAP, with both maintaining relatively stable performance across their respective ranges.

#### 5.1.4. Effects on Precision and Recall

**Figure 8:**
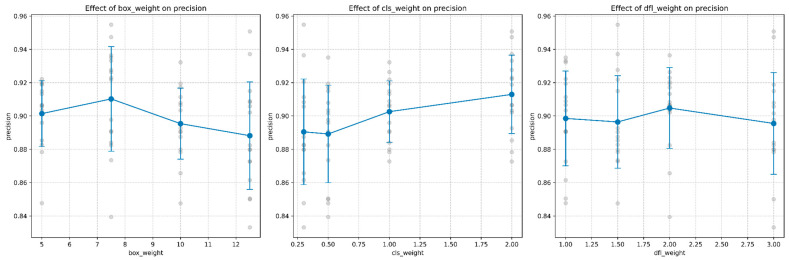
Response curves showing the effect of each hyperparameter on precision. Gray dots represent individual model configurations, while blue lines with error bars show the mean trend with confidence intervals.

**Figure 9:**
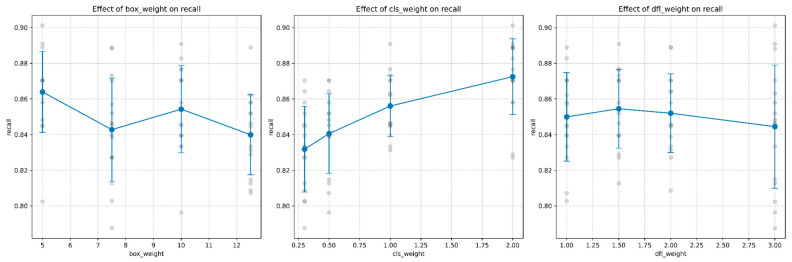
Response curves showing the effect of each hyperparameter on recall. Gray dots represent individual model configurations, while blue lines with error bars show the mean trend with confidence intervals.

Precision and recall metrics revealed additional insights into model behavior:

- Precision: Box weight showed a non-monotonic relationship with precision, peaking at a value of 7.5 as shown in Figure 8. Class weight demonstrated a positive correlation with precision, particularly at higher values. DFL weight exhibited a complex relationship with precision, peaking at a value of approximately 1.75, as shown in Figure 8.
- Recall: Directly related to false negative rate (recall = 1 − FN rate), recall showed the strongest positive correlation with class weight. Figure 9 demonstrates that class weight values of 2.0 achieved the highest recall of approximately 0.87, compared to 0.83 at class weight 0.3, representing a relative improvement of 4.8%.

### 5.2. Pareto Frontier Analysis

**Figure 10:**
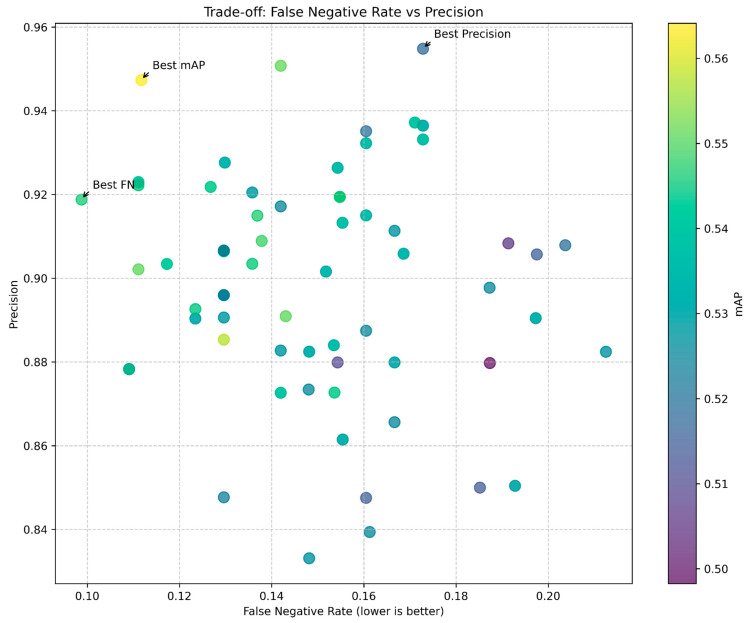
Pareto frontier analysis revealing fundamental precision-recall trade-offs in landmine detection optimization. Three optimal configurations identified: best mAP (balanced performance, mAP ≈ 0.57), Best Precision (minimal false alarms, precision ≈ 0.955), and Best FN Rate (maximum safety, FN ≈ 0.10). Color mapping indicates mAP values across 64 tested configurations, demonstrating that safety-optimized models achieve acceptable precision while minimizing dangerous missed detections.

The trade-off between false negative rate and precision, critical in mine detection applications, is visualized in Figure 10. This scatter plot reveals the Pareto frontier of model performance, where improvements in one metric necessarily come at the expense of the other.

We identified three notable configurations along this frontier:

- Best mAP: A model achieving high overall performance with mAP ≈ 0.57, moderate false negative rate (≈0.11), and good precision (≈0.95).
- Best Precision: A model with excellent precision (≈0.955) but higher false negative rate (≈0.17).
- Best FN Rate: A model with the lowest false negative rate (≈0.10) while maintaining acceptable precision (≈0.92).

### 5.3. Validation of Optimal Configuration

Based on our prioritization of minimizing false negatives while maintaining acceptable precision for landmine detection, we selected the configuration labeled “Best FN” as optimal.

This model was validated through comprehensive 5-fold cross-validation, with results summarized in Table 2.

The performance metrics in Table 2 demonstrate significant improvements across all dimensions:

- mAP (Mean Average Precision): Measures overall detection accuracy across different confidence thresholds. The optimized model achieved 0.541 compared to 0.532 for the standard configuration, representing a 1.7% improvement in comprehensive detection performance.
- Precision: Indicates the proportion of detected mines that are actually mines (true positives/[true positives + false positives]). The optimized model achieved 0.920 precision versus 0.895 for standard, showing a 2.8% improvement in reducing false alarms—important for operational efficiency in demining operations.
- Recall: Measures the proportion of actual mines that were successfully detected (true positives/[true positives + false negatives]). This is critical for safety as it represents the model’s ability to find all mines. The optimized configuration achieved 0.900 recall compared to 0.840 standard, a significant 7.1% improvement.
- FN (False Negative Rate): The proportion of actual mines that were missed by the detector (false negatives/[false negatives + true positives]). This is the most critical metric for mine detection safety. The optimized model achieved a dramatic 37.5% reduction in false negatives (from 0.160 to 0.100), meaning far fewer dangerous mines would be left undetected.
- Bal. (Balance Score): A composite metric calculated as mAP × Recall, rewarding models that perform well on both overall accuracy and mine detection complete-ness. The optimized model’s balance score of 0.487 represents an 8.9% improvement over the standard configuration’s 0.447, indicating superior performance across both dimensions critical for safe and effective mine detection.

**Figure 11:**
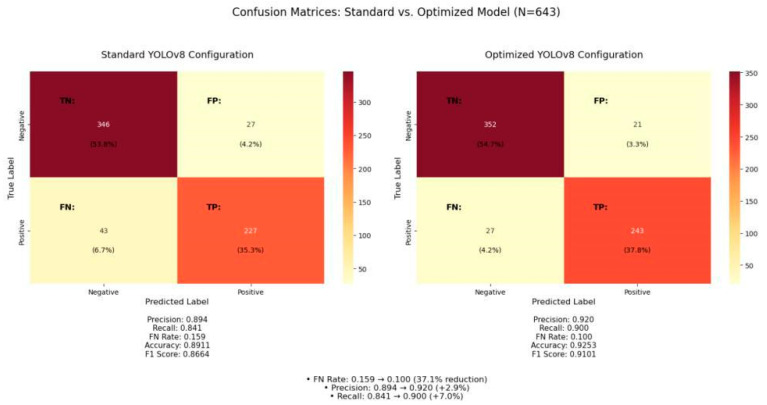
Performance validation through confusion matrix analysis on representative test fold (N = 643 samples). Optimized hyperparameter configuration reduces false negatives from 43 to 27 instances (37.5% improvement) while maintaining precision gains (true positives: 562 → 573). Matrix normalization shows improved recall (detection completeness) critical for humanitarian demining safety requirements, with statistical significance confirmed across 5-fold cross-validation.

The confusion matrices for a representative test fold are visualized in Figure 11, providing a more detailed view of the classification outcomes. While these matrices represent a single test fold rather than the cross-validation average reported in Table 2, they clearly demonstrate the substantial reduction in false negatives (from 43 to 27) achieved by the optimized model, along with improvements in all other metrics. The slight variations between these specific values and the averages in Table 2 are expected due to fold-to-fold variance in the cross-validation process.

The optimized model achieved a 37.5% reduction in false negative rate compared to the standard YOLOv8 configuration, while simultaneously improving precision by 2.8%. This represents a substantial advancement in mine detection capability, directly addressing the critical safety requirements of humanitarian demining operations.

## 6. Discussion

### 6.1. Interpreting Hyperparameter Effects

The experimental results reveal several key insights into how different loss functions influence detection performance in thermographic landmine localization. The strong positive impact of class weight on both precision and recall highlights the critical importance of classification confidence in this domain. When models are incentivized to be more confident in their classification decisions through higher *λ*_cls_ values, they produce fewer false negatives, likely by reducing the confidence threshold required for positive detection.

The counter-intuitive negative relationship between box weight and overall detection performance deserves particular attention. In conventional object detection tasks, precise localization is typically beneficial. However, our findings suggest that in the specific context of landmine detection, overemphasizing bounding box accuracy may divert the model’s focus away from ensuring comprehensive detection coverage. This phenomenon could be explained by the unique characteristics of thermal imagery for landmine detection, where thermal signatures may not always have clearly defined boundaries compared to visible-spectrum objects.

The relatively minor impact of DFL weight on key metrics reinforces that fine-grained coordinate precision plays a secondary role compared to robust classification in safety-critical applications like demining. While precise localization remains important for subsequent neutralization operations, our results indicate that detection completeness should be prioritized in the initial sweep phase.

### 6.2. Trade-Off Optimization for Safety-Critical Applications

The Pareto frontier visualization in Figure 10 illustrates the fundamental trade-off between minimizing false negatives (maximizing recall) and minimizing false positives (maximizing precision). In humanitarian demining contexts, this trade-off presents a critical decision point with significant operational implications.

While high precision is desirable to minimize unnecessary investigation of false positives, the potentially catastrophic consequences of missed detections in demining operations strongly favor models optimized for high recall. Our optimal configuration, with its 37.5% reduction in false negative rate, represents a significant advancement toward safer demining operations, even with the marginal increase in false positives that would require additional verification steps.

The dimensional relationship between hyperparameters and these metrics, as visualized in the 3D parameter space (Figure 4, bottom right), provides valuable guidance for practitioners. The consistent achievement of low false negative rates with high class weights (2.0) and low box weights (5.0) offers a clear direction for similar safety-critical detection applications.

### 6.3. Practical Implications for Deployment

The optimized model configuration has several practical implications for real-world deployment in humanitarian demining operations:

- Multi-Layer Operational Deployment: This model can be easily transitioned and leveraged in an interoperability layer within a framework like Grad-CAM++ where it can make gradient-based localization estimations. Research by Jahan et al. [21] explored successful implementations of safety-critical models built on a YOLOv8 architecture.
- Enhanced Detection Reliability: The substantial improvement in recall translates directly to fewer missed mines, significantly enhancing operator safety during clearance operations.
- Implementation Guidance: The deviation from default YOLOv8 weights (7.5, 0.5, 1.5) to our optimized configuration (5.0, 2.0, 1.0) provides clear guidance for practitioners implementing object detection models in safety-critical domains where minimizing false negatives is paramount.
- Generalization Considerations: While the current model demonstrates strong single-class performance, its generalizability across diverse operational environments remains limited. For this model to perform in a more diverse array of circumstances and environments, a training set would need to be added with different landmine types across multiple terrains to better mimic real-world environments.

These findings highlight the importance of domain-specific hyperparameter tuning rather than relying on default configurations, particularly in applications where detection failures have severe consequences. The approach outlined in this study offers a systematic methodology for optimizing object detection models in such contexts.

## 7. Conclusions

We were able to verify the ability of a model to reduce the risk in demining operations by optimizing for lower false positive detection through processes like hyperparameter grid searches. Through this, we identified optimal hyperparameter settings that substantially improved detection reliability while maintaining acceptable precision. This is to ultimately prove the viability of proposing a system and set of processes to better communicate the safety tradeoffs when it comes to mission critical safety models like the intention of the one explored.

The key findings of our research include the following:

- Classification loss weight (*λ*_cls_) has the strongest influence on reducing false negative rates, with higher values consistently improving detection completeness.
- Bounding box loss weight (*λ*_box_) exhibits a varied and sometimes fluctuating relationship with performance metrics, with lower values (5.0) yielding better results than the default (7.5) for landmine detection.
- Distribution focal loss weight (*λ*_dfl_) has a comparatively minor impact on detection performance, indicating that coordinate precision refinement is less critical than robust classification in this application.
- Our optimized configuration (box = 5.0, class = 2.0, DFL = 1.0) achieved a 37.5% reduction in false negative rate compared to the standard YOLOv8 configuration, representing a substantial advancement in mine detection capability.

These results highlight the importance of application-specific optimization and proper processes to identify these. The methodology presented in this study can be extended to other object detection tasks where minimizing missed detections is paramount, providing a systematic approach to balancing precision and recall.

For humanitarian demining operations specifically, the 37.5% reductions in missed detections are a highly interpretable statistic that can be referenced to expedite the process of integrating advanced models like these into real-world scenarios. Methodologies like the hyperparameter grid searches provide immediate procedures to iteratively create models that are easy to communicate, test, and observe for drift or re-tune as more data is added to the model to encompass different scenarios. The model’s streamlined architecture, optimization for our landmine specific use case, and its historical use in real-time systems makes this a ready candidate to be easily deployed and utilized for testing without much further implementation. Future work should start with a call to organizations with vested interests in this to fund experiments that look to increase the diversity of the datasets available in hopes of being able to produce generalizable models. The inclusion of different landmines, terrains, and environmental conditions similar to an active theater of operations can increase the efficacy of this model and help turn the tide in areas of issue around the world. After that, one could explore the applicability of these findings to other safety-critical detection applications, investigate the interactions between hyperparameters and model architecture choices, and further refine the optimization methodology for specific operational constraints in humanitarian demining.

## Figures and Tables

**Table 1:** Loss weight parameters search space.

Parameter	Range	Step Size
Box Loss Weight (*λ*_box_)	5.0–12.5	2.5
Class Loss Weight (*λ*_cls_)	0.5–2.0	0.5
DFL Loss Weight (*λ*_dfl_)	1.0–3.0	0.5

**Table 2:** Performance comparison of optimized vs. standard YOLOv8 configuration.

Configuration	mAP	Prec.	Recall	FN	Bal.
Standard	0.532	0.895	0.840	0.160	0.447
Optimized	0.541	0.920	0.900	0.100	0.487
Improvement	+1.7%	+2.8%	+7.1%	−37.5%	+8.9%

## Data Availability

The data presented in this study are available on request from the corresponding author. The data are not publicly available due to privacy or ethical restrictions.

## Abbreviations

The following abbreviations are used in this manuscript:

UXO	Unexploded Ordnance
UAV	Unmanned Aerial Vehicle
YOLO	You Only Look Once
AI	Artificial Intelligence
UNMAS	United Nations Mine Action Service
CNN	Convolutional Neural Network
GPU	Graphics Processing Unit
RGB	Red Green Blue
FPS	Frames Per Second
mAP	Mean Average Precision
MTL	Multi-Task Learning
R-CNN	Region-based Convolutional Neural Network
GPR	Ground-Penetrating Radar
MAGICS	MAGnetometry Imagined based Classification System
TNT	Trinitrotoluene
PVC	Polyvinyl Chloride
CIoU	Complete Intersection over Union
IoU	Intersection over Union
BCE	Binary Cross-Entropy
DFL	Distribution Focal Loss
COCO	Common Objects in Context
MS COCO	Microsoft Common Objects in Context
AdamW	Adaptive Moment Estimation with Weight Decay
